# Utility of ^18^F-FDG PET/CT in Detecting Spinal Drop Metastases from Pineal Gland Tumors

**DOI:** 10.22038/AOJNMB.2024.74259.1518

**Published:** 2024

**Authors:** Kabilash Dhayalan, Harish Goyal, Pradap Palanivelu, Dhanapathi Halanaik

**Affiliations:** Department of Nuclear Medicine Jawaharlal Institute of Postgraduate Medical Education and Research (JIPMER), Puducherry, India

## Abstract

Pineal gland tumors are significant despite being rare (<1%) among all brain tumors. Germ cell tumors are the most common among the pineal gland tumors. Often affecting young adults, pineal gland germ cell tumors are hard to diagnose due to different symptoms and potential spread. But they rarely show leptomeningeal spread and extracranial metastases. Other differentials include primary tumors of the pineal region, Pineal gliomas, and metastases. The leptomeningeal spread of these tumors has not been studied so far. Conventional radiological imaging modalities are routinely used to diagnose and evaluate these tumors. We report a case here showing a pineal gland tumor with leptomeningeal spread detected by ^18^F-FDG PET/CT. Our case shows how pineal gland tumors can behave unusually and how ^18^F-FDG PET/CT can be crucial for accurately assessing the extent of the disease in the body to provide effective treatment. This case report illustrates the rare type of spread of pineal gland tumor and how ^18^F-FDG PET/CT helps detect this rare type of metastasis, thereby helping in prognostication and deciding further treatment of the patient.

## Introduction

 Pineal gland tumors are rare and constitute less than 1% of all primary brain tumors. Germ cell tumors (GCTs) are the most frequently encountered type among these tumors. The pineal gland, located deep within the brain, regulates sleep-wake cycles and produces melatonin. When a tumor develops in this gland, it can interfere with these functions and lead to symptoms that vary based on the tumor's size and location (1- 3). 

 Given pineal gland tumors' rarity and diverse histology, each case should be approached individually, considering the tumor's specific characteristics and behavior. The ability of ^18^F-FDG PET/CT imaging provides a comprehensive evaluation of the disease, aiding in developing appropriate treatment strategies and monitoring therapeutic response (4-5).

 Drop metastases refer to the dissemination of CNS tumor cells along the cerebrospinal fluid pathways, resulting in the settlement of tumor deposits in intraspinal locations. Certain types of brain tumors, including pineal gland tumors, often exhibit this pattern of metastasis. It is worth noting that intraspinal metastases are also associated with other intracranial tumors, such as medulloblastoma, ependymoma, pineoblastoma, astrocytoma, lymphomas, choroid plexus papillomas, retinoblastomas (6- 8).

 Detecting drop metastases in the spinal cord and FDG uptake in the primary lesion and satellite lesions in the brain through ^18^F-FDG PET/CT imaging provides valuable information about the extent and distribution of the tumor. This information is critical for accurate staging, treatment planning, and monitoring of treatment response.

## Case report

**Figure 1:**
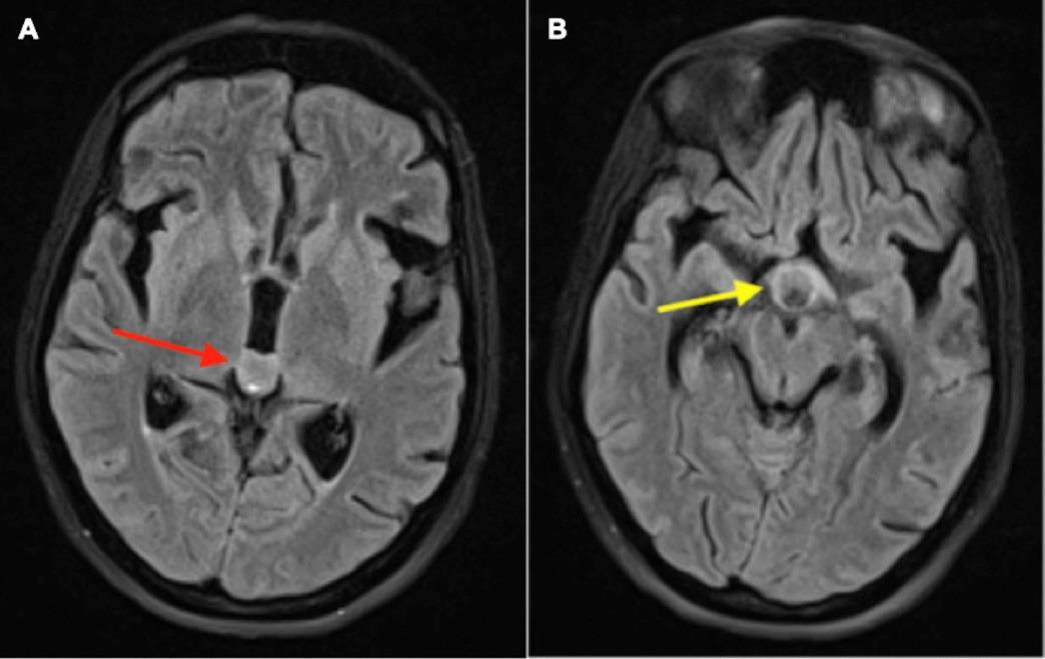
Brain MRI images showing mildly bulky pineal gland (**A**), bulky pituitary infundibulum (**B**)

 A 30-year-old woman complained of headaches, vomiting, and missed periods for three months. She also experienced progressive paraparesis over six weeks, leading to bilateral lower limb paralysis. An ophthalmological evaluation revealed papilledema and a relative afferent pupillary defect. The patient underwent brain Magnetic Resonance Imaging (MRI) to examine potential intracranial pathology. The brain MRI revealed two significant findings: a bulky pineal gland and involvement of the pituitary infundibulum (Figure 1: T2-weighted axial images). These findings are consistent with a pineal gland tumor or other pathologies affecting the pineal region. The involvement of the pituitary infundibulum suggests potential extension or compression of the tumor into nearby structures. Elevated serum alpha-fetoprotein (AFP) levels, beta-human chorionic gonadotropin (β-hCG), and CA-125 tumor markers provided additional clues. Obtaining a biopsy from pineal gland tumors can be challenging due to the location and associated risks.

**Figure 2:**
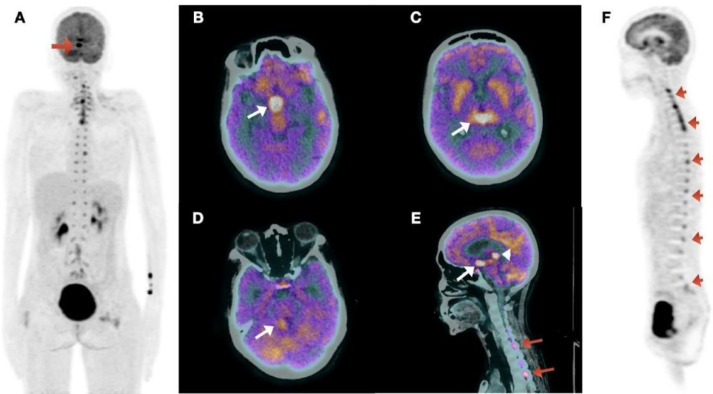
18F-FDG PET/CT: Increased tracer uptake in intracranial and paravertebral regions (**A**: MIP image). On fused axial images there is increased tracer uptake in suprasellar region (**B**) and pineal gland region (**C**) and cerebellar vermis region (**D**). Sagittal fused image (**E**) reveals increased tracer uptake in pineal gland (**arrow head**) and suprasellar regions (**arrow**), and cervical nerve roots (**red arrow**). On sagittal PET image there is increased tracer uptake in cervical spinal cord, lumbar, and sacral nerve roots (**F**)

**Figure 3:**
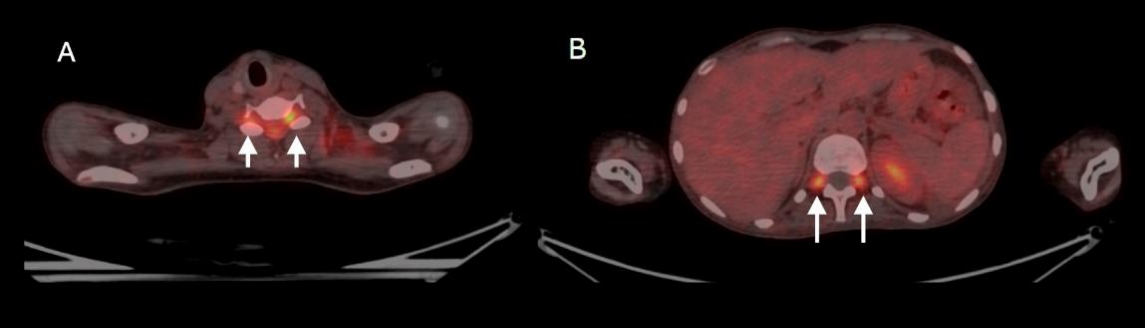
Transaxial fused PET/CT images at cervical level (**A**) and lumbar level (**B**) showing increased FDG uptake in the intervertebral foramina (**white arrow**)

Subsequently, the patient underwent ^18^F-FDG PET/CT imaging. The findings indicated focal FDG uptake in the pineal gland, suprasellar region, cerebellar vermis, fourth ventricle, cervical spinal cord, and multiple nerve roots (Figure 2). The Maximum Intensity Projection (MIP) image (Figure 2; A) showed increased FDG uptake in the brain's pineal gland region. Axial hybrid PET/CT images demonstrated increased FDG uptake in the suprasellar region (Figure 2; B), pineal gland region (Figure 2; C), and cerebellar vermis (Figure 2; D). The sagittal hybrid PET/CT image (Figure 2; E) revealed increased FDG uptake in the pineal gland, suprasellar regions, and cervical nerve roots (arrowheads). The sagittal PET image (Figure 2; F) showed increased FDG uptake in the cervical spinal cord and lumbar and sacral nerve roots (Figure 3). 

 Based on the combination of symptoms, neurological deficits, elevated tumor markers, and the imaging features of ^18^F-FDG PET/CT and brain MRI, a possible differential diagnosis is a pineal gland tumor, particularly a pineal germ cell tumor. These tumors can cause obstructive hydrocephalus, leading to increased intracranial pressure and the potential for metastatic spread, including drop metastases to the spinal cord. However, no definite histopathological diagnosis could be made as the patient expired due to ventilatory complications before a complete diagnosis was made.

## Discussion

 Tumors of the pineal region are rare, accounting for less than 1% of intracranial tumors, with most being pure germinomas. The other differential include primary tumors of the pineal region, Pineal gliomas, and metastases. Imaging techniques such as head and spine MRI and measuring levels of α-fetoprotein and β-human chorionic gonadotropin help diagnose intracranial germ cell tumors (GCTs). While spinal and extracranial metastases are infrequent, they may be missed on regional imaging (9-10), including only brain MRI and brain CECT. Spinal drop metastases can be evaluated by spinal CSF analysis or spinal MRI. 

 Their role in the diagnosis of leptomeningeal spread is still unclear. In one study conducted in the pediatric population, the false-negative rate of MRI was reported as 4%, and CSF-cytology was 16% (11). ^18^F-FDG PET/CT imaging is valuable in evaluating and characterizing pineal gland tumors, particularly in identifying satellite lesions and spinal cord metastases. It complements the assessment conducted through AFP and β-hCG tests. Since biopsy can be challenging, metabolic imaging helps provide insights into tumor characteristics. By offering a comprehensive evaluation in a single session, ^18^F-FDG PET/CT assists in determining the appropriate treatment approach based on an accurate tumor burden and distribution assessment. It is especially beneficial in detecting metastases of intracranial germinomas, including those in the spine and outside the brain. This imaging technique aids in evaluating the status of the disease and guiding treatment decisions. While most germ cell tumors exhibit increased ^18^F-FDG uptake, mature teratomas show minimal or no uptake. Pineal gland germ cell tumors rarely metastasize to the spine, and only a few cases are documented. 

 However, the histopathological diagnosis of this case was not arrived, which is a major limitation of this report. However, spinal radiotherapy and chemotherapy have demonstrated effectiveness as salvage treatments for such patients (12- 14).

 In summary, whole-body ^18^F-FDG PET/CT imaging is crucial in visualizing and characterizing pineal germ cell tumors and their metastases, including drop metastases. ^18^F-FDG PET/CT helps in detecting this leptomeningeal spread better than other conventional imaging modalities and hence it plays an important role in evaluating these patients. Detecting leptomeningeal spread is crucial in deciding proper treatment plan for the patient.
